# Aging‐associated changes in hippocampal glycogen metabolism in mice. Evidence for and against astrocyte‐to‐neuron lactate shuttle

**DOI:** 10.1002/glia.23319

**Published:** 2018-03-01

**Authors:** Dominika Drulis‐Fajdasz, Agnieszka Gizak, Tomasz Wójtowicz, Jacek R. Wiśniewski, Dariusz Rakus

**Affiliations:** ^1^ Department of Molecular Physiology and Neurobiology University of Wroclaw, Sienkiewicza 21 Wroclaw 50‐335 Poland; ^2^ Department of Proteomics and Signal Transduction Max‐Planck‐Institute of Biochemistry, Am Klopferspitz 18 Martinsried 82152 Germany

**Keywords:** aging, energy metabolism, hippocampal proteome

## Abstract

Lactate derived from astrocytic glycogen has been shown to support memory formation in hippocampi of young animals, inhibiting it in old animals. Here we show, using quantitative mass spectrometry‐based proteomics, immunofluorescence, and qPCR that aging is associated with an increase of glycogen metabolism enzymes concentration and shift in their localization from astrocytes to neurons. These changes are accompanied with reorganization of hippocampal energy metabolism which is manifested by elevated capacity of aging neurons to oxidize glucose in glycolysis and mitochondria, and decreased ability for fatty acids utilization. Our observations suggest that astrocyte‐to‐neuron lactate shuttle may operate in young hippocampi, however, during aging neurons become independent on astrocytic lactate and the metabolic crosstalk between the brain's cells is disrupted.

## 1 INTRODUCTION

During the past decade a growing body of evidence has accumulated that energy metabolism of astrocytes is a crucial player in regulation of neuronal network properties. It turned out that inhibition of glycogen breakdown and lactate release from astrocytes inhibits memory consolidation in chicks (Gibbs, Anderson, & Hertz, 2006) and disrupts memory formation and Long Term Potentiation (LTP; a molecular mechanism of memory formation) in murine hippocampi (Suzuki et al., 2011). However, all the mentioned results were obtained using young or juvenile animals (Gibbs et al., 2006; Suzuki et al., 2011).

In contrast, just recently, we have demonstrated that the inhibition of glycogen breakdown in astrocytes of old animals significantly elevates LTP magnitude in hippocampal slices and affects the mode of dendritic spines maturation (Drulis‐Fajdasz et al., 2015). In other words, inhibition of glycogen degradation in hippocampi of old animals prevents aging‐associated defects in memory formation.

To obtain a deeper insight into age‐associated changes we investigated the expression and localization of proteins of energy metabolism in hippocampi of young (1 month) and middle‐aged (12–13 months) mice using immunofluorescent methods and a quantitative proteomics technique—total protein approach (TPA), which delivers information about the absolute (expressed in moles or moles per/mg of protein) abundance/concentration of proteins in studied samples (Wiśniewski, Hein, Cox, & Mann, 2014).

We present evidence that physiological aging process is associated with substantial changes in the expression and cellular localization of energy metabolism proteins in the hippocampal formation. Notably, glycogen metabolism enzymes expression and localization undergo significant changes which emphasizes the role of glycogen in age‐related decline of cognitive functions and points to proteins involved in the regulation of glycogen metabolism in hippocampus as a targets of an anti‐aging therapy.

Glycogen degradation is strictly associated to the fate of glucose described by the hypothesis called astrocytes‐to‐neurons lactate shuttle (ANLS). As a matter of fact, the term “ANLS” is used for description of two different but very closely related physiological processes. The most popular interpretation of the ANLS says that the majority of glucose in brain is utilized by astrocytes which convert it into pyruvate which, after reduction to lactate, is released from astrocytes and serves neurons to produce energy in the process of oxidative phosphorylation (Pellerin & Magistretti, 1994; Pellerin & Magistretti, 2012; Serres, Bezancon, Franconi, & Merle, 2004). The second one, which we name here LTP‐ANLS, operates during the memory (LTP) formation (Dringen, Gebhardt, & Hamprecht, 1993; Drulis‐Fajdasz et al., 2015; Suzuki et al., 2011) and is involved in regulation of miniature postsynaptic potentials (Kaczor, Rakus, & Mozrzymas, 2015; Mozrzymas, Szczęsny, & Rakus, 2011). The latter mechanism seems to be well documented although the manner/molecular mechanism in which astrocytic glycogen‐derived lactate supports LTP and miniature postsynaptic currents is still under investigation.

Results of our analyses of young hippocampi are roughly in line with the ANLS hypothesis, both in the “classical” interpretation and as the LTP‐ANLS. However, our studies on middle‐aged animals do not support the existence of such a shuttle. They suggest that in aging hippocampus, neurons become independent to astrocytic metabolism: they have elevated concentrations of glycolytic enzymes and do not consume astrocyte‐derived lactate but they are able to release the monocarboxylates. Moreover, aging neurons accelerate synthesis of glycogen metabolism enzymes and glutamine synthase. Thus, the age‐related changes in astrocyte and neurons proteome may explain the controversies that aroused hot disputes about the existence and the significance of the ANLS (Dienel, 2017; Lundgaard et al., 2015; Steinman, Gao, & Alberini, 2016).

Regardless of the controversies, our results show that the ability of aging hippocampal neurons to oxidize glucose in glycolysis becomes higher than in young ones. This is accompanied by significant elevation of mitochondrial energy metabolism and rearrangement of mitochondrial network.

The data presented here provides the most comprehensive quantitative description of energy metabolism proteins expression in murine hippocampi during aging. It also demonstrates that, because of basically different organization of metabolism in hippocampi of young and adult/old animals, entirely different approaches to the treatment of young and aging patients suffering from medical conditions associated with energy metabolism in brain must be taken into account.

## 2 METHODS

### 2.1 Animals and tissue preparation

The experiments were performed on 2 groups of male C57BL/10J mice: young (P30, *n* = 6) and aged (12 months, *n* = 6). Animals were anesthetized with isoflurane and decapitated. Hippocampi were isolated in ice‐cold buffer containing (in mM): NaCl 87, KCl 2.5, NaH2PO4 1.25, NaHCO_3_ 25, CaCl_2_ 0.5, MgSO_4_ 7, glucose 25, sucrose 75; pH 7.4. The left hippocampi from each animal were analyzed using quantitative proteomics, the right ones were used for immunofluorescence. All the procedures were approved by the local Ethical Commission and every effort was made to minimize the number of animals used for the experiments.

### 2.2 Hippocampal lysates

Hippocampi were homogenized immediately after isolation in buffer containing 0.1 M Tris/HCL, 2% SDS, 50 mM DTT, pH 8.0 and incubated 5 min at 99°C. Samples were cooled in liquid nitrogen and stored in −20°C until proteomic analysis. Total protein was determined by measuring tryptophan fluorescence as described in Wiśniewski and Gaugaz (2015).

### 2.3 Protein digestion and peptide fractionation

Protein lysates were processed in the 30K filtration units (Millipore, Darmstadt, Germany) using the MED‐FASP protocol (Wiśniewski & Mann, 2012). Peptides were quantified as described previously (Wiśniewski, 2013).

### 2.4 LC – MS/MS analysis

Analysis of the peptide mixtures was performed in Orbitrap instrument (Thermo Fisher Scientific, Waltham, Massachusetts, USA) as described previously (Wiśniewski & Mann, 2012). Aliquots containing 5 μg of total peptides were chromatographed on a 50 cm column with 75 µm inner diameter packed C_18_ material (Dr. Maisch GmbH, Ammerbuch‐Entringen, Germany). Peptide separation was carried out at 300 nl/min for 75 min using a two‐step acetonitrile gradient, 5%–40% over the first 60 min and 40%–95% for the following 15 min. The temperature of the column oven was 55°C.

The mass spectrometer operated in data‐dependent mode with survey scans acquired at a resolution of 50,000 at m/z 400 (transient time 256 ms). Up to the top 15 most abundant isotope patterns with charge ≥ +2 from the survey scan (300–1650 m/z) were selected with an isolation window of 1.6 m/z and fragmented by HCD with normalized collision energies of 25. The maximum ion injection times for the survey scan and the MS/MS scans were 20 and 60 ms, respectively. The ion target value for MS1 and MS2 scan modes was set to 3 × 10^6^ and 10^5^, respectively. The dynamic exclusion was 25 s and 10 ppm.

The mass spectrometry data have been deposited to the ProteomeXchange Consortium via the PRIDE partner repository (Vizcaíno et al., 2013) with the dataset identifier: PXD006815.

### 2.5 Proteomic data analysis

The spectra were searched using MaxQuant software. All raw files were searched together in a single MaxQuant run. For the chymotryptic peptides generated from the GluC‐pre‐cleaved material a mixed Chymotryptic/tryptic specificity was set. A maximum of two missed cleavages was allowed. Carbamidomethylation was set as fixed modification. The “matching between the runs “option was used. The maximum false peptide and protein discovery rate was specified as 0.01. Protein abundances were calculated using the “total protein approach” (TPA) (Wiśniewski et al., 2014; Wiśniewski & Rakus, 2014). The calculations were performed in Microsoft Excel.

### 2.6 Immunofluorescence

Hippocampi were fixed in 4% PFA and embedded in paraffin. 4‐μm sections were stained as described before (Wiera et al., 2012). Primary and secondary antibodies with the used dilutions are listed in Supporting Information, Table S1. Tissue sections were examined with a FluoView 1000 confocal microscope (Olympus, Tokyo, Japan) at 20× and 60× magnification at 2,048 × 2,048 picture resolution.

### 2.7 Sample collection for quantitative real‐time PCR

RT‐PCR, experiments were performed on two groups of female C57BL6 mice: young (P30, *n* = 4) and middle‐aged (13 month‐old, *n* = 4). Animals were anesthetized with isoflurane and decapitated and hippocampi were isolated and sliced using a McIlwain tissue chopper (TedPella, Redding, California, USA; slices were 300 µm thick), as described previously (Ting, Daigle, Chen, & Feng, 2014). The slices were placed under stereoscope (Optatech, Warszawa, Poland) and CA1 hippocampal layers were dissected with a tip of a needle (0.3 × 8 mm) and the region of stratum pyramidale of each slice was absorbed directly to TRI Reagent‐containing (Applied Biosystems, Waltham, Massachusetts, USA) syringe. From each animal, 5–10 dissected stratum pyramidale samples were collected (Supporting Information, Figure S1).

### 2.8 RNA isolation and cDNA synthesis

From every individual hippocampal slice, the total mRNA within CA1 stratum pyramidale region was prepared separately according to the manual (Applied Biosystems, Waltham, Massachusetts, USA). Reverse transcription was performed with total mRNA according to procedure enclosed for iScript cDNA Synthesis Kit (Bio‐Rad, Hercules, California, USA). Obtained cDNA samples were used for qRT‐PCR reactions. All procedures were performed with RNA‐free equipment (e.g., getting rid of RNase by Ambion's RNase*Zap*, Thermo Fisher, Waltham, Massachusetts, USA) and DEPC‐treated reagents.

### 2.9 Real‐time PCR

For detection of mRNA for metabolic genes commercially available TaqMan probes (Applied Biosystems, Waltham, Massachusetts, USA) dedicated to mouse tissue were used (see Supporting Information, Table S2). The level of mRNA for glycogen synthase (*Gys1*) was used as reference (Kim et al., 2014). In contrast to *Gys1*, our proteomic data revealed that the expression of all commonly used in qRT‐PCR referential genes (Boda, Pini, Hoxha, Parolisi, & Tempia, 2009) changed during aging or they were expressed at very low levels (Supporting Information, Table S3).

Gene expression analysis was performed with iTag Universal Probes Supermix (Bio‐Rad, Hercules, California, USA) and standard thermal cycling conditions using the LightCycler 480 Instrument (50 cycles of amplification). Each qRT‐PCR reaction was performed in triplicate and we tested nine genes simultaneously on one plate to minimize manual handling errors. The relative gene expression level was estimated using Pfaffl algorithm and Roche LightCycler 480 Analysis Software (Basel, Switzerland). At least 20 “Young” and 20 “Old” qRT‐PCR reactions for single gene were analyzed and mean relative expression level versus *Gys1* reference gene (Real Time PCR R‐score) was calculated. To visualize distribution of RT‐PCR R‐scores between samples, results were presented in the form of cumulative histograms (Figure 8a).

### 2.10 Statistical analysis

All results are presented as mean ± *SEM* unless otherwise stated. The statistical analysis was performed using Student's *t* test preceded by Fisher *F* test. We used non‐paired Student's *t* test for comparisons between any two experimental groups. Image analysis was performed using Fiji open‐source software (Schindelin et al., 2012) or Cell ^F software (Olympus Soft Imaging Solutions GmbH, Tokyo, Japan). The statistical analyses were performed using SigmaPlot 11 software (Systat Software). Statistical significance of differences is indicated by asterisks: *p <* *.05, **p < .01, ***p < .001.

## 3 RESULTS

### 3.1 Proteomic analysis

Although steady progress has been made over recent decades, the quantitative description of basic metabolic pathways in brain is still far from being complete. Up to date, almost all studies on brain energy proteome were performed using mass spectrometry analysis of spots cut from 2D‐PAGE after their densitometric measurement (for review see VanGuilder & Freeman, 2011). This makes these analyses partial and not really quantitative in terms used by biology and chemistry (for review see Gizak & Rakus, 2016). The newest, gel‐free, studies of hippocampal proteome have been dedicated to investigate Alzheimer's disease‐related changes and do not provide information about energy metabolism changes during aging (e.g., Neuner, Wilmott, Hoffmann, Mozhui, and Kaczorowski, 2017). In contrast, the deepest gel‐free proteomic analysis of brain aging described by Walther and Mann (2011) has been performed using adult and very old mice, respectively, 5 and 26 months‐old.

**Figure 1:**
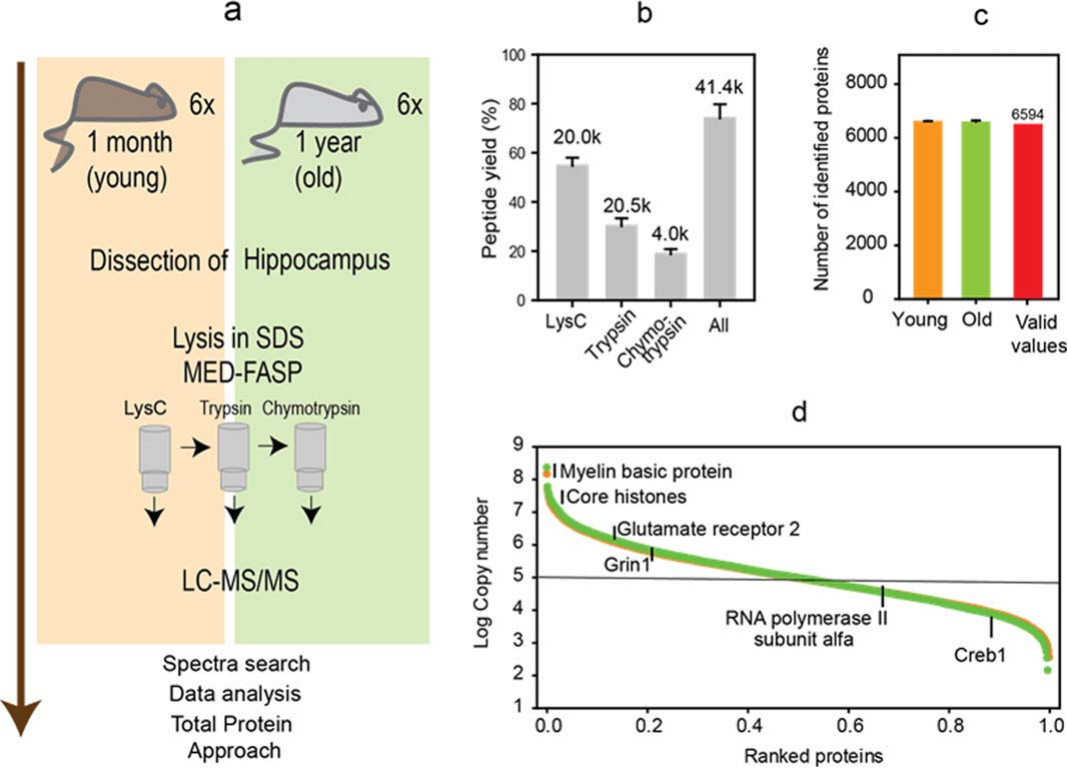
Proteomic analysis of hippocampi from young and middle‐aged mice. (a) Proteomic workflow. Hippocampi were isolated from 6 animals per group. The tissue lysates were processed by MED‐FASP procedure with three steps of enzymatic digestion. Peptides were analyzed by LC‐MS/MS. The spectra were searched using MaxQuant software. Proteins were quantified by means of Total Protein Approach. (b) Protein to peptide conversion and peptide analysis. The numbers above the columns show the average number of peptides identified from each digest (*k*, values are in thousands). (c) Number of proteins matching the peptides identified in the mass spectrometric analysis. 6,594 proteins were identified in at least 9 of 12 samples (valid values) and were used for the statistical analysis. (d) Distribution of protein copy numbers across the identified proteins [Color figure can be viewed at http://wileyonlinelibrary.com]

In this article, we provide the first quantitative description of young (1 month‐old) and middle‐aged (12 month‐old) mice hippocampi (Figure 1a). The proteomic method involved the MED‐FASP procedure for protein digestion (Figure 1a) with a 3‐step protein cleavage with LyC, trypsin and chymotrypsin. We achieved 80% conversion of protein to peptides (Figure 1b). This high yield was the prerequisite for the label free quantitative analysis. The analysis of six biological replicates of young and aged hippocampi allowed identification of average 6,700 proteins per sample (Supporting Information, Table S4; Figure 1c). 6,594 proteins were measured in at least 9 (from 12) samples. The data covers six orders of magnitude of protein abundance (Supporting Information, Table S4; Figure 1d).

**Figure 2:**
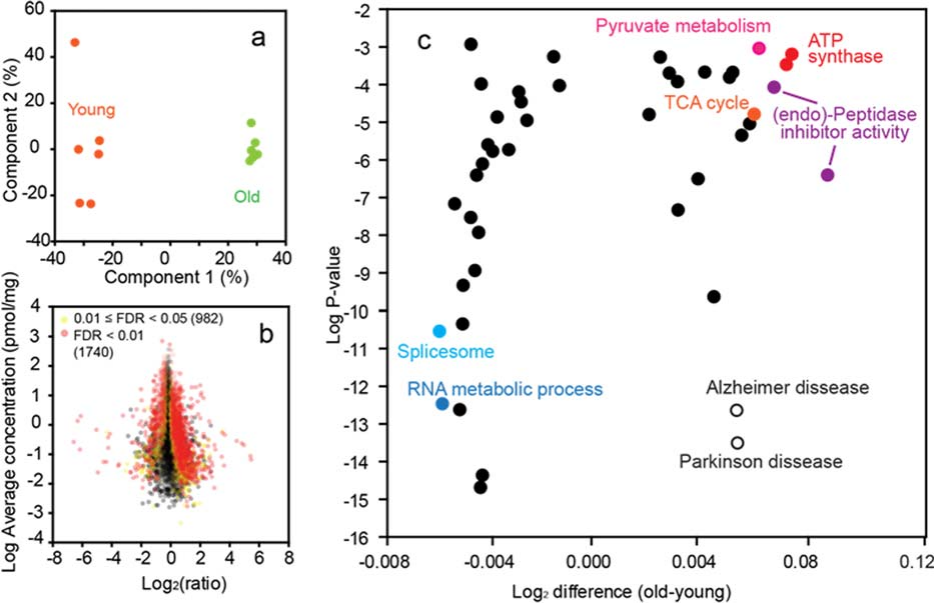
Statistical analysis of the proteomic data. (a) Principal component analysis of the protein concentration values of 6,594 protein (valid values). Missing values were imputed. (b) Heat map: Distribution of protein ratios versus protein concentrations. Proteins with significantly changed titers are shown in yellow (.01 ≤ FDR < .05) and red (FDR < .01). Numbers in the parentheses show the number of proteins in each group. (c) Identification of significantly changed protein groups matching gene ontology or KEGG terms (complete data set is in Supporting Information, Table S4) [Color figure can be viewed at http://wileyonlinelibrary.com]

Principal component analysis revealed a clear separation of the quantitative proteome data between the groups of young and aged animals (Figure 2a). *t* Test analyses identified 1,740 proteins with significantly changed titers at false discovery rate (FDR) < .01 (Figure 2b). Additionally, 982 proteins were changed at FDR < .05. The statistical analyses allowed identification of 44 significantly altered protein groups matching “Gene Ontology” or “KEGG” categories (Supporting Information, Table S4; Figure 2c). Age related changes encompass upregulation of basic energy metabolism pathways including TCA cycle, pyruvate metabolism and ATP synthase, augmentation of endoproteinase inhibitors and upregulation of Alzheimer's and Parkinson's disease‐associated proteins. In contrast, the machinery of protein synthesis and fatty acids transport was downregulated. Here, we focus on the changes in energy metabolism pathways and a detailed discussion of these changes is given below.

### 3.2 Enhanced glycogen metabolism in aged hippocampal neurons

**Figure 3:**
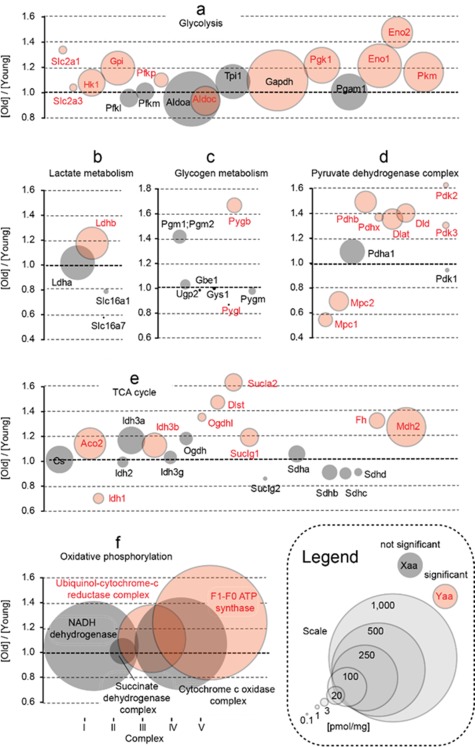
Changes in the energy metabolism processes upon aging. Plots show ratios of protein concentrations in old vs. young hippocampi. The size of bubbles is proportional to an average protein concentration. Proteins with significantly changed concentrations are highlighted in red. (a) Glucose transporters and glycolysis. (b) Lactate metabolism. (c) Glycogen metabolism. (d) Pyruvate dehydrogenase complex. (e) Krebs cycle (TCA). (f) Oxidative phosphorylation (the plot bubbles refer to the sum of all subunits in each complex) [Color figure can be viewed at http://wileyonlinelibrary.com]

**Figure 4:**
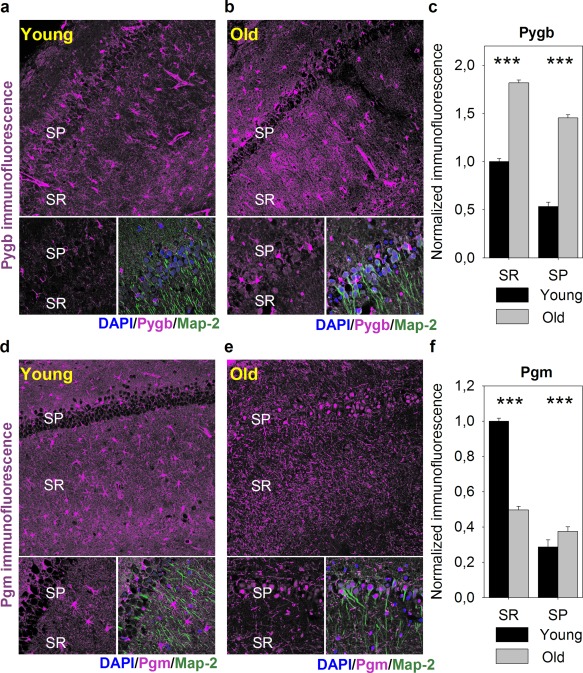
Cellular localization of glycogen phosphorylase (Pygb) and phosphoglucomutase 1 (Pgm) in hippocampus is altered with aging. (a–b) Exemplary confocal images of Pygb immunofluorescence (magenta) distribution within hippocampal CA1 region in young (a) and aged (b) animals acquired at low (obj. 20×, upper panel) and high magnification (obj. 60×, bottom panels). Localization of neuronal somata, dendrites and nuclei was revealed with antibodies against microtubule‐associated protein 2 (Map2, green) and DAPI (blue), respectively. Abbreviations: SP, stratum pyramidale; SR, stratum radiatum. (c) Quantification of Pygb immunofluorescence in hippocampal slices of young (black bars) and old animals (gray bars). Note, that aging promotes an increase in Pygb immunofluorescence in both hippocampal layers. (d and e) Exemplary confocal images of Pgm immunofluorescence (magenta) distribution within hippocampal CA1 region in young (d) and aged (e) animals acquired in conditions described in a and b. (f) Quantification of Pgm immunofluorescence in hippocampal slices of young (black bars) and old animals (gray bars). All immunofluorescence was normalized to values obtained within stratum radiatum of young animals. Note that with age, Pgm immunofluorescence declines in dendritic region of hippocampal CA1 region, but rises within neuronal somata. Asterisks indicate a statistically significant difference (*p* < .001) [Color figure can be viewed at http://wileyonlinelibrary.com]

As shown in Figure 3a–c, protein levels for several enzymes involved in glycolysis, lactate and glycogen metabolism were upregulated with age. Especially significant, about 1.7‐fold increase in protein concentration was observed in the whole brain homogenates for the brain isoform of glycogen phosphorylase (Pygb), the main protein of glycogen degradation (Supporting Information, Table S5; Figure 3c). Importantly, Pygb cellular distribution underwent changes during aging. While in young animals, it was present predominantly in putative astrocytes (Figure 4a,c), in middle‐aged mice, it was located both in astrocytes and in neurons (Figure 4b,c). Our previous studies have shown that inhibition of glycogen breakdown in astrocytes had an opposite effect on the LTP magnitude in young and middle‐aged rats: in the young group it disrupted LTP maintenance whereas in the aged one it appeared to favor LTP formation (Drulis‐Fajdasz, 2015). Therefore, we conclude that the capability to synthesize and degrade glycogen is higher in hippocampi of middle‐aged than young mice (Figure 3c; Figure 4a–c; Supporting Information, Table S5). Moreover, the amount of mRNA for Pygb was significantly elevated in neurons dissected from striatum pyramidale of aged animals (Figure 8). Remarkably, similar age‐dependent changes in the localization were observed for phosphoglucomutase isoforms, Pgm1/2 (Figure 4d–f), the enzyme directing glucose (precisely: glucose‐6‐phosphate) from glycolysis to glycogenogenesis. In young animals, Pgm1/2 was located mainly in astrocytes while in middle‐aged hippocampi, it was evenly distributed between the cells (Figure 4d–f). The total concentration of Pgm1/2 in hippocampus measured by TPA was 1.4 times higher in older animals, however, the changes were not statistically significant (Supporting Information, Table S5; Figure 3c).

Since inhibition of glycogen degradation in young hippocampi (attributed solely to astrocytes) diminish LTP formation and inhibition of glycogen phosphorylase in aged ones (associated with astrocytes and neurons) rescues LTP, it strongly suggest that neuronal glycogen degradation intermediates are deleterious for neuronal functions (Magistretti & Allaman, 2007).

### 3.3 Alterations in glycolysis – disruption of the ANLS shuttle and glutamine cycle during aging

**Figure 5:**
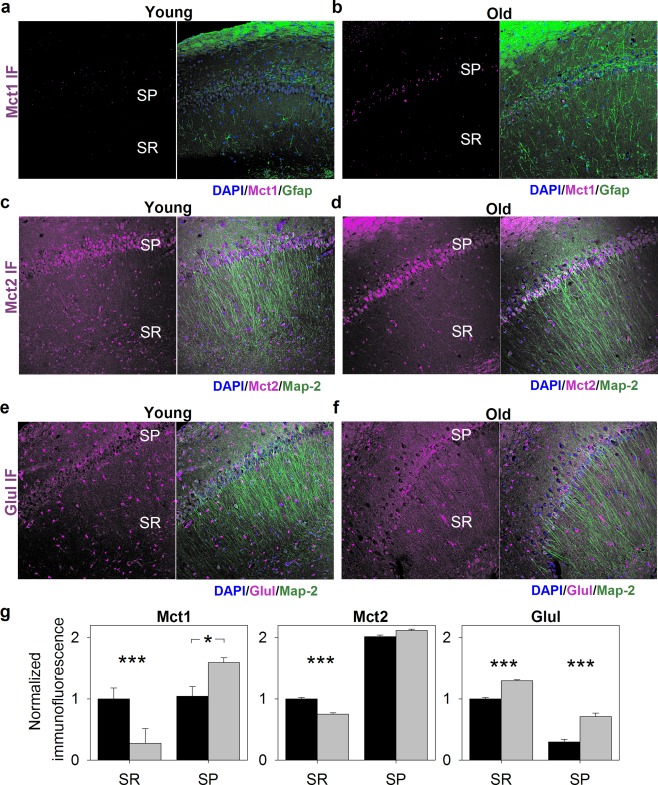
Cellular distribution of monocarboxylate transporters (Mct) and glutamine synthetase (Glul) in the hippocampus of young and middle‐aged (“Old”) animals. (a–f) Exemplary confocal images of immunofluorescence distribution for Mct1 (a–b, magenta), Mct2 (c and d, magenta) and Glul (e and f, magenta) acquired at low magnification (obj. 20×) from slices of young (left panels) and aged animals (right panels). Localization of neurons, astrocytes and cellular nuclei was revealed with antibodies against microtubule‐associated protein 2 (Map2, green), glial fibrillary acidic protein (GFAP) and DAPI (blue), respectively. Abbreviations: SP, stratum pyramidale; SR, stratum radiatum. (g) Quantification of immunofluorescence for Mct1 (left panel), Mct2 (middle panel), and Glul (right panel). All immunofluorescence was normalized to values obtained within stratum radiatum of young animals. Note, that aging promotes an increase in Glul immunofluorescence in both SP and SR hippocampal layers. In contrast, Mct1 and Mct2 were downregulated in the area on neuronal dendrites (SR) and Mct2 distribution was shifted towards neuronal somata (SP). Asterisks indicate a statistically significant difference (*p <* *.05, ***p <* *.001). IF, immunofluorescence [Color figure can be viewed at http://wileyonlinelibrary.com]

ANLS should be characterized by higher expression of glycolytic enzymes and proteins of glucose uptake and lactate release in astrocytes. Our data showed that in young hippocampi, monocarboxylate transporter Mct1 (Slc16a1) regarded as crucial for the release of lactate from cells (for review see Bergersen, 2007), was randomly distributed in a dot‐like manner within CA1 layers (Figure 5a). However, in middle‐aged animals, the Mct1 was found within stratum pyramidale (in putative neuronal cells) (Figure 5b,g) which suggests that hippocampal neurons of middle‐aged animals may release monocarboxylates, e.g. lactate.

The level of fluorescent signal associated with Mct4, the transporter involved in lactate extrusion from astrocytes (Bergersen, 2007), was very low and randomly dispersed within hippocampus (data not shown). Probably, Mct4 is expressed at extremely low level, especially in young mice, as we could not detect it using the MS technique, and it does not play any significant role in brain lactate transport. The Mct2 protein (Slc16a7) which is thought to be associated with lactate uptake (Bergersen, 2007) both in young and old mice, was localized mainly in neurons (SP) and aging did not affect the fluorescence signal from this region (Figure 5c,d,g). In contrast to SP, the fluorescence signal in stratum radiatum was significantly reduced in middle‐aged mice when compared to the young ones (Figure 5c,d,g). These changes were accompanied with almost 1.7‐fold decrease (*p* = .052) in the total Mct2 concentration as measured with TPA method.

Proteomically, aging significantly elevated the level of almost all glycolytic and glucose uptake proteins, however, the increase was not very high and it was in the range of 10%–20% (Supporting Information, Table S5; Figure 3). We also observed an increase in the titer of Pfk regulatory proteins (Pfkp and TIGAR) but the changes were not statistically significant (Supporting Information, Table S5). These results corroborate the findings of Freeman, VanGuilder, Bennett, and Sonntag, (2009) who have found that in rat brain, aging correlated with enhanced expression of Pfk. However, in contrast to these studies, we did not observe a chaotic dysregulation of glycolysis but a well‐orchestrated increase in the level of practically all glycolytic proteins. In contrast to these proteins, among proteins associated directly with lactate metabolism and transport only the concentration of lactate dehydrogenase B was significantly modified by aging (Supporting Information, Table S5; Figure 3b).

**Figure 6:**
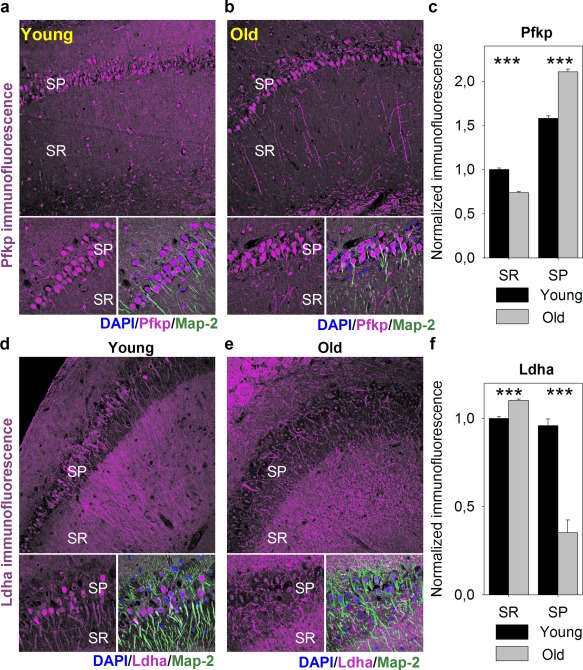
Cellular distribution of phosphofructokinase platelet form (Pfkp) and lactate dehydrogenase (Ldha) in the hippocampus of young and middle‐aged (“Old”) animals. (a and b) Exemplary confocal images of Pfkp immunofluorescence (magenta) distribution within hippocampal CA1 region in young (a) and aged (b) animals acquired at low (obj. 20×, upper panel) and high magnification (obj. 60×, bottom panels). Localization of neuronal somata, dendrites and nuclei was revealed with antibodies against microtubule‐associated protein 2 (Map2, green) and DAPI (blue), respectively. Abbreviations: SP, stratum pyramidale; SR, stratum radiatum. (c) Quantification of Pfkp immunofluorescence in hippocampal slices of young (black bars) and old animals (gray bars). Note, that aging promotes an increase in Pfkp immunofluorescence in neuronal somata (SP), unlike region of dendritic trees (SR). (d and e) Exemplary confocal images of Ldha immunofluorescence (magenta) distribution within hippocampal CA1 region in young (d) and aged (e) animals acquired in conditions described in a and b. (f) Quantification of Ldha immunofluorescence in hippocampal slices of young (black bars) and old animals (gray bars). All immunofluorescence was normalized to values obtained within stratum radiatum of young animals. Note, that aging is associated with a shift in Ldha cellular distribution from SP towards SR. Asterisks indicate a statistically significant difference (*p *<* *.001) [Color figure can be viewed at http://wileyonlinelibrary.com]

Interestingly, our studies revealed that a slight but statistically significant elevation of Pfkp titer in middle‐aged hippocampi (Supporting Information, Table S5; Figure 3a) was correlated with a decrease of Pfkp‐related fluorescence in putative astrocytes (the SR region) and its increase in neurons (Figure 6a–c) which was reflected by elevation of neuronal mRNA for this isoform (Figure 8). This indicates that aging increases capability of neurons to degrade glucose in glycolysis making them independent on astrocytic glucose‐derived metabolites.

Another manifestation of aging was different localization of several energy metabolism enzymes in neurons. While Mct2 and Pfkp changed their localization from neurites to neuronal cell bodies (Figure 5c,d,g and Figure 6a–c), Ldha‐related fluorescence in neuron cell bodies became weaker in aged hippocampi (Figure 6d–f). Interestingly, in neuronal cell bodies of young animals, a very strong Ldha‐related signal was detected (Figure 6d) suggesting that neurons are able to produce (and release) lactate even in young hippocampi. In contrast, the changes in mRNA level for *Ldha* (Figure 8) did not reflect the observed changes in protein level measured both by TPA and by immunofluorescence analysis. This indicates the lack of simple correlation between mRNA production and protein abundance for Ldha in neurons.

**Figure 7:**
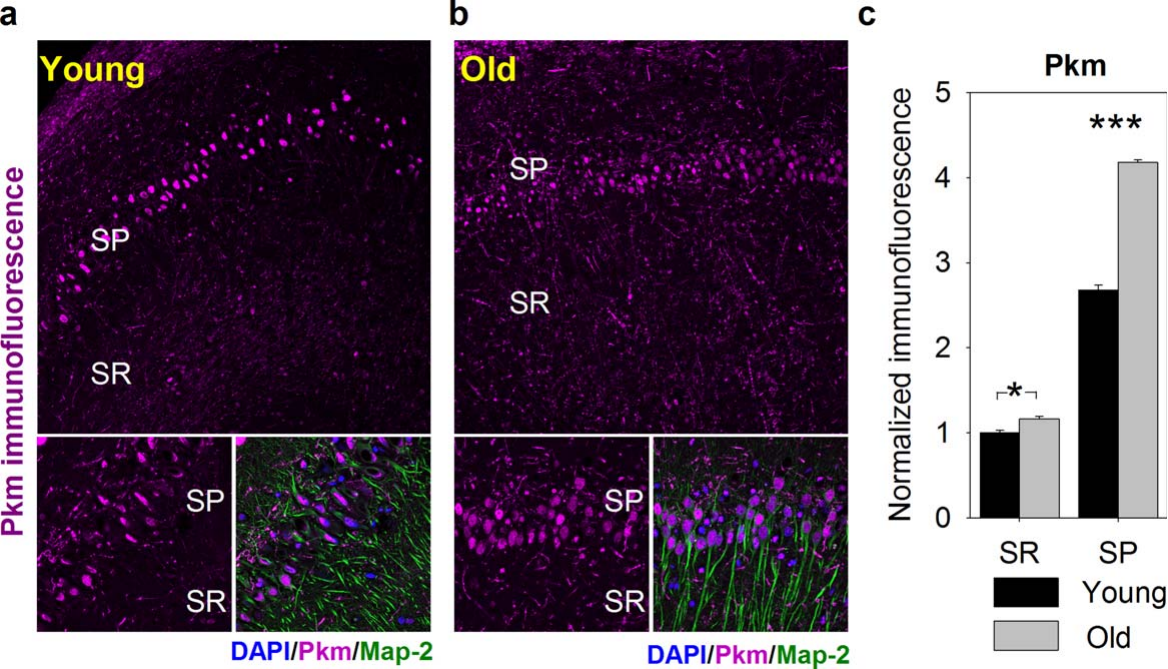
Cellular distribution of pyruvate kinase (Pkm) in the hippocampus of young and middle‐aged (“Old”) animals. (a and b) Exemplary confocal images of Pkm immunofluorescence (magenta) distribution within hippocampal CA1 region in young (a) and aged (b) animals acquired at low (obj. 20×, upper panel) and high magnification (obj. 60×, bottom panels). Localization of neuronal somata, dendrites and nuclei was revealed with antibodies against microtubule‐associated protein 2 (Map2, green) and DAPI (blue), respectively. Abbreviations: SP, stratum pyramidale; SR, stratum radiatum. (c) Quantification of Pkm immunofluorescence in hippocampal slices of young (black bars) and old animals (gray bars). Note that with age, Pkm immunofluorescence is significantly upregulated in both SR and SP layers. Asterisks indicate a statistically significant difference (*p <* *.05, ***p <* *.001) [Color figure can be viewed at http://wileyonlinelibrary.com]

**Figure 8:**
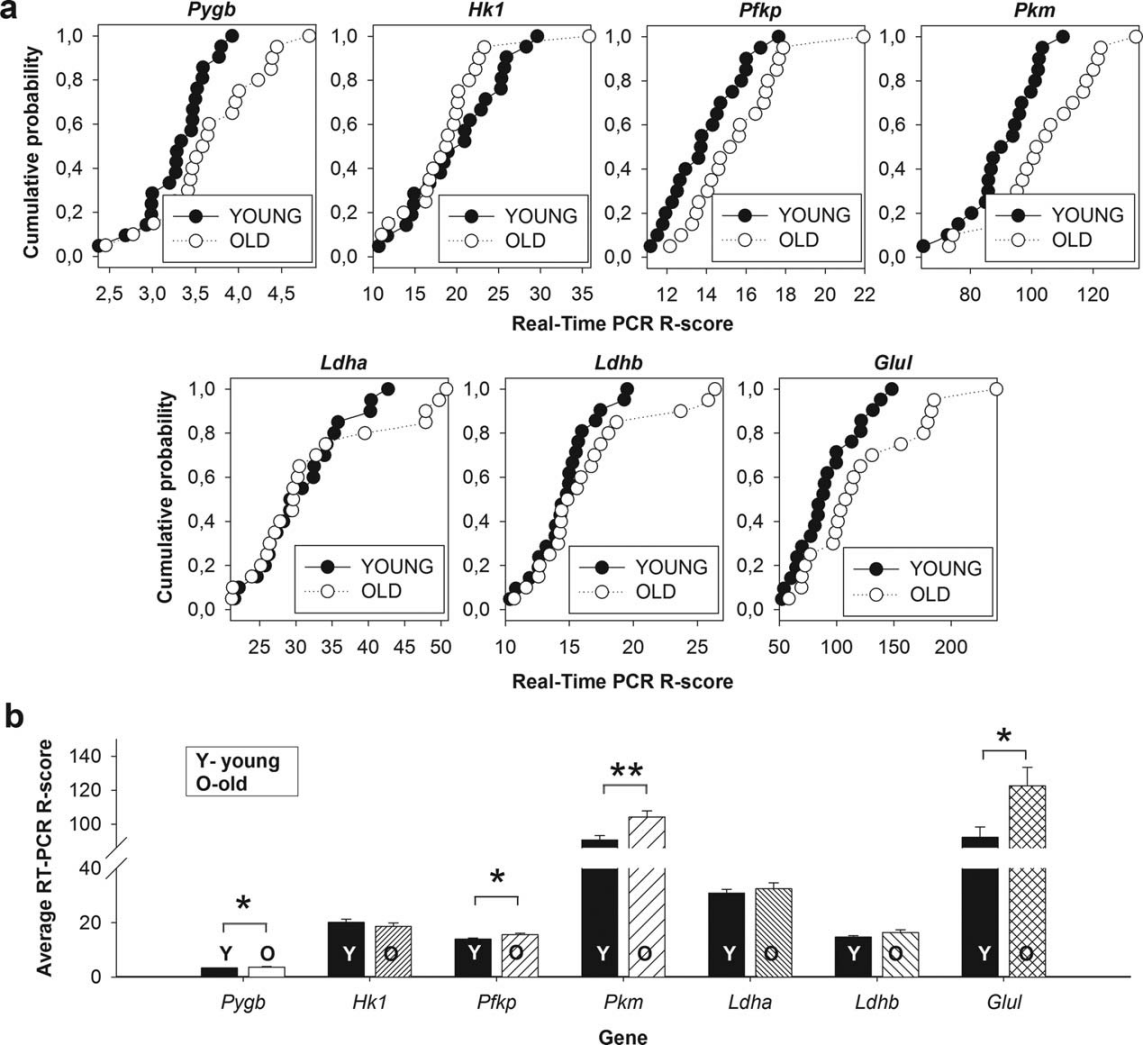
Upregulation of gene expression for metabolic enzymes with aging. (a) Cumulative histograms for gene expression assays for selected enzymes in brain tissue samples collected from hippocampal CA1 region (stratum pyramidale) of young (black circles) and aged animals (white circles). Note the right‐shift of the distribution of RT‐PCR scores with aging in majority of investigated genes. See Section 2 for details. Abbreviations: *Pygb*, glycogen phosphorylase; *Hk1*, hexokinase 1; *Pfkp*, phosphofructokinase platelet form; *Pkm*, pyruvate kinase; *Ldha/b*, lactate dehydrogenase a/b; *Glul*, glutamine synthetase. (b) Quantification of the results shown in (a). Asterisks indicate a statistically significant difference (*p < .05, **p < .01)

In line with hypothesis of the ANLS, the localization of Hk1 (Supporting Information, Figure S2a–c) in young animals was predominantly astrocytic (in SR) and aging had now significant effect on Hk1 distribution in neurons and astrocytes. In contrast to Hk, the last regulatory enzyme of glycolysis—Pkm, localized mainly in neuronal cell bodies (Figure 7a–c) and aging significantly elevated the level of its protein and mRNA in neurons (Figure 3, Figure 8).

Taken together, the distribution of glycolytic enzymes in young hippocampi is roughly in line with the hypothesis of the ANLS. However, changes in the localization of carbohydrate metabolism proteins during aging suggest disruption of metabolic astro‐neuronal ‘syncytial’ network and transition from the astrocyte‐based to the neuron‐located metabolism of glucose.

Another manifestation of the interruption of the astrocyte‐neuron cross‐talk are changes in glutamine synthetase (Glul) localization. While in young mice, Glul was localized predominantly in astrocytes, aging correlated with significant reduction of astrocytic Glul and increase of its mRNA and protein in neurons (Figure 5e–g, Figure 8). As it has been shown previously, neurons in co‐culture with astrocytes express only small amount of Glul while in the absence of astrocytes, the expression of Glul in neurons is significantly increased (Fernandes, Dringen, Lawen, & Robinson, 2010; Mamczur et al., 2015).

### 3.4 Aging alters mitochondrial energy metabolism

Neurons, as highly active cells, rely on mitochondrial function. It has been shown that aging induces changes that include decline in neuronal glucose uptake and decrease of electron transport chain activity and hence, in ATP production (for review see Yin, Sancheti, Liu, & Cadenas, 2016). In contrast, a proteomic study using isolated brain mitochondria has revealed that the bioenergetic function of aged mitochondria was preserved (Stauch, Purnell, Villeneuve, & Fox, 2015).

The results of proteomic studies presented here are roughly in line with the latter study (Stauch et al., 2015). For several proteins of the tricarboxylic acid cycle (TCA) and oxidative phosphorylation (OXPHOS) we observed slight but statistically significant increase in their titers during aging (Supporting Information, Table S5). This elevation of individual proteins concentrations was accompanied with an increase in the total proteins amount of TCA pathway and OXPHOS complex V (ATP synthase complex) (Supporting Information, Table S5; Figure 3e,f).

The main substrates for ATP production: Acetyl‐CoA and α‐ketoglutarate, may be synthesized, respectively, from pyruvate and fatty acids (FA) and from glutamine, however, in brain interstitial fluid, the titer of glutamine is much lower than in cerebrospinal fluid (e.g., Dolgodilina et al., 2016) and thus, this amino acid is not presumed to play any important role in TCA supplementation.

In accordance to the increased catabolic capacity of aged hippocampi, we found that all components of pyruvate dehydrogenase complex (Pdh) were more abundant in old hippocampi (Supporting Information, Table S5; Figure 3d). Unexpectedly, the abundance of mitochondrial pyruvate carrier proteins (Mpcs), indispensable for pyruvate uptake by mitochondria, was strongly reduced (Supporting Information, Table S5; Figure 3d). The discrepancy between the significant increase in Pdh components titer and decrease of Mpcs capacity to transport pyruvate may be associated with an elevated ability of aged hippocampi to inhibit Pdh complex by pyruvate dehydrogenase kinases, Pdk1–3, which concentration was significantly higher in middle‐aged mice (Supporting Information, Table S5; Figure 3d).

In contrast to the elevated concentration of enzymes of the catabolic pathways, the concentration of fatty acid‐binding proteins (Fabps), crucial for FA targeting to β‐oxidation, was much lower in aged animals (Supporting Information, Table S5). This implies that during aging mitochondrial ATP production by hippocampal cells becomes more dependent on glucose catabolism than on FAs.

### 3.5 Age‐associated reorganization of mitochondrial network

**Figure 9:**
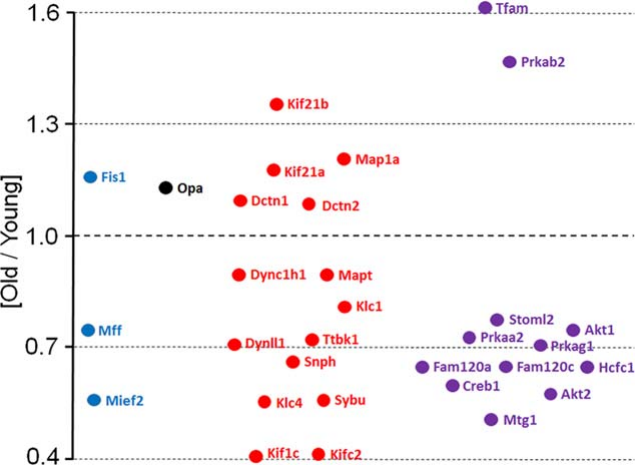
Age‐related changes in concentration of proteins regulating mitochondrial motility and biogenesis. The plot shows average ratios of protein concentrations in young vs. aged (“old”) animal brains. Only the proteins that significantly (*p* < .05) changed their concentration with age are presented. Blue – proteins engaged in mitochondrial fission, black – fusion, red – motility, purple – biogenesis [Color figure can be viewed at http://wileyonlinelibrary.com]

ATP production by mitochondria depends both on the concentration of proteins of metabolic machinery (transporters of substrates, TCA, OXPHOS) and on the organization and dynamics of mitochondrial network. In the hippocampi, we did not observe a significant alteration in the total titer of proteins involved in mitochondria biogenesis (Supporting Information, Table S5). However, we found that among these biogenesis‐related proteins that did significantly change their titers with age (12 out of 23 proteins) the aging‐related decrease applied to majority of them (i.e., 10) (Supporting Information, Table S5; Figure 9). A meaningful exception was the rising titer of mitochondrial transcription factor A (Tfam), the protein required for mitochondrial DNA transcription and maintenance of proper level of mtDNA (Larsson et al., 1998). This implies the rising need for mitochondrially‐encoded proteins and increasing efforts to ensure the adequate level of non‐damaged mtDNA in older animals.

In contrast, in hippocampi of older mice, we found about 21% decrease in sum of mitochondrial fission protein and ∼28% increase in sum of proteins executing fusion (Supporting Information, Table S5; Figure 9). Although majority of changes in the individual proteins were statistically insignificant, together they seem to point to a growing tendency towards mitochondrial fusion in old hippocampi. Fusion allows for exchange of mtDNA between healthy and dysfunctional mitochondria. Its aging‐related intensification might suggest an increased requirement for mitochondrial bioenergetics optimization arising from e.g. accumulation of mtDNA defects. Presence of such defects might be substantiated by significant rise in the proteolysed form of a fusion protein OPA1—s‐OPA1, in older mice (Supporting Information, Table S5; Figure 9). It was demonstrated that intensified proteolytic cleavage of OPA1 occurs in cells with impaired mitochondrial respiration (Möpert et al., 2009). The shift from mitochondrial fission to elongation might spare the organelles from autophagy and maximize ATP production.

Both fusion and fission are also prerequisite to proper positioning of mitochondria in a cell. The prevalence of fusion in older mice suggests reduced mitochondrial dynamics and disturbance in precise positioning of the organelles in neuronal processes, which might result in defects in synaptic homeostasis. Perturbation in mitochondrial dynamics is observed in some neurological disorders, like Parkinson's, Alzheimer's and Huntington's disease, and it correlates with decreased energy supply for synaptic activity (Chen & Chan, 2009).

Analysis of proteins engaged in mitochondrial transport in neurons (motor‐adaptor proteins; based on Sheng & Cai, 2012) revealed statistically significant differences in titers of only 15 out of 50 of these proteins between young and old hippocampi (Supporting Information, Table S5; Figure 9). Majority of these differences (11 out of 15) were in favor of the young structures. However, there was no significant difference in the total motor‐adaptor protein amount between young and old hippocampi, even though it is well known that mitochondrial transport gradually declines with age, leading to energy deficits and inability of in neurons to regenerate. Moreover, in older animals, we observed a significantly lower titer of syntaphilin—a specific static anchor of axonal mitochondria. This protein is supposed to peak in the adult brain and reduce motility of axonal mitochondria, thus, decreasing energy supply to axons (Zhou et al., 2016).

In contrast, in older animals, there was a significant decrease in titer of syntabulin. This protein drives mitochondrial transport to neuronal processes, which is crucial for maintenance of synaptic functions (Ma, Cai, Lu, Sheng, & Mochida, 2009). In agreement with this decrease, we found increased signal of cytochrome c in neuronal somata in old mouse hippocampal slices (Supporting Information, Figure S2d–f).

This might suggest that although in old hippocampus, the reduction of syntaphilin‐mediated mitochondrial anchoring in axons facilitates return of damaged mitochondria to the somata, in the same time, transport of the new, functional organelles to axons is significantly reduced.

Together, these results might point to some perturbations of mitochondria positioning in neuronal processes in aged hippocampi. Thus, the age‐related decline in memory formation might result not from reduction of ATP production in mitochondria but from disturbance of transport of the organelles to the sites of high ATP demands.

## 4 DISCUSSION

The results presented here revealed that aging correlates with entire reorganization of energy metabolism machinery, both in astrocytes and in neurons.

Glycogen degradation in brain (precisely: in astrocytes) is strictly associated with the fate of glucose described by the hypothesis called astrocytes‐to‐neurons lactate shuttle (ANLS), according to which astrocytic glycogen‐derived lactate supports neuronal metabolism and plasticity.

Up to date, there is no convincing hypothesis of how this mechanism works. The “energetic” explanation is that the lactate being a substrate for neuronal oxidative phosphorylation may directly, via increase in ATP synthesis (for review see Obel et al., 2012), and/or indirectly, via postsynaptic changes in NADH, potentiate NMDA signaling (Yang et al., 2014). In contrast, it cannot be excluded that lactate plays a role of an autocrine signaling molecule (Morland et al., 2015). Our study support the “energetic” hypothesis, but only in young mice. In these animals, we observed distribution of crucial enzymes/proteins of energy metabolism among astrocytes and neurons that was in agreement with the ANLS hypothesis.

However, we found that the molar ratio of Pyg to Gys (a glycogen degradation to glycogen synthesis enzyme) in hippocampus is very high and similar as in white skeletal muscle fibers (Rakus, Gizak, & Wiśniewski, 2016), which are suited for the rapid glycogen degradation. This suggests that episodes of glycogen degradation in astrocytes are rapid and thus, the short‐time local increase in lactate may be sufficient to stimulate neuronal energy production in OXPHOS. By analogy to white skeletal muscle fibers, it might be hypothesized that the ANLS is highly active and significantly participates in neuronal metabolism predominantly during a high cell activity, e.g. during memory formation, when glycogen stores in astrocytes are mobilized to support neuronal OXPHOS (“LTP‐ANLS”).

Furthermore, we found that concentration of glycogen metabolism enzymes significantly increased during aging. We also observed aging‐associated relocation of Pygb, a regulator of glycogen degradation, and Pgm1, the enzyme directing glucose to glycogen synthesis, to neurons. Previously, we have demonstrated that in contrast to young rats, the inhibition of glycogen degradation in old animals improves neuronal plasticity and reverts aging‐associated changes in LTP formation (Drulis‐Fajdasz, 2015). This have led us to made an assumption that the increased level/activity of Pygb in neurons of hippocampi is the main cause of aging‐associated decline in memory formation.

However, in brain, glycogen is stored mostly in astrocytes since accumulation of this polymer in neurons stimulates apoptosis and is a hallmark of several neurodegenerative disorders (for example Lafora progressive myoclonus epilepsy). Thus, under physiological conditions neurons suppress the storage via laforin‐malin complex. Laforin alone is a glycogen storage‐promoting enzyme but in complex with malin, it stimulates proteasomal degradation of glycogen synthase (Solaz‐Fuster et al., 2008). We found more than five‐fold, statistically significant elevation of laforin titer in old mice hippocampi (Supporting Information, Table S5). Together with the increase in Pgm in neurons, this may point to enhanced glycogen synthesis in old animals, which would be deleterious to neurons. Thus, the observed changes of Pygb and glucose degradation enzymes titers and localization enhancing neuronal ability to degrade glycogen to pyruvate and release lactate into extracellular space might be a necessary anti‐neurodegenerative mechanism operating in aging hippocampus. In the same time, a diminished glycolytic capacity of astrocytes, together with a decreased amount of transporters suited to lactate secretion (Mct1) would balance the rise in neuronal production of this compound, and astrocytes would use lactate produced by neurons to feed mitochondrial metabolism. Mangia, Simpson, Vannucci, and Carruthers, (2009) proposed a model according to which neurons might transfer lactate to astrocytes (NALS, neuron‐to‐astrocyte lactate shuttle) in specific conditions, however, this hypothesis lacked experimental proof.

In contrast, lactate is protective in many neurodegenerative diseases. Its elevated concentrations protect against Aβ‐related neuronal toxicity (Newington, Harris, & Cumming, 2013). Thus, even if lactate is not produced to be an energy source to astrocytes, it could be an important neuroprotective agent. In this context, however, it is hard to explain results of our previous studies demonstrating that inhibition of glycogen phosphorylase (associated with astrocytes and neurons) rescues LTP in old animals. It cannot be excluded that this inhibition is beneficial only in a short term, until the deleterious effects of neuronal glycogen accumulation take their toll. In such case, the anti‐neurodegenerative protection of neurons would be prioritized against memory formation in aging brain.

Along with the increased glycogen metabolism in neurons, we observed the age‐dependent elevation of glycolytic and oxidative metabolism capacity of these cells. It is unclear if metabolic changes precede or are an effect of changes in the structure of hippocampus. Recently, we have shown that astrocytes cultured in the absence of neurons have lower expression of regulatory enzymes of glycolysis while in neurons cultured alone, the expression is basically higher than in the astrocyte‐neuron co‐culture (Mamczur et al., 2015). This finding has been corroborated, on the level of mRNA, by (Hasel et al., 2017). In brain, the spatial separation of astrocytes from neurons may result from increased volume of extracellular matrix as it has been demonstrated by (Végh et al., 2014). In line with this, we found a significant elevation of extracellular proteins concentration in the middle‐aged hippocampi (Supporting Information, Table S5).

It has been shown that lack of the proper astrocyte‐neuron cross‐talk is reflected by change in Glul expression in neurons and astrocytes (Fernandes et al., 2010; Mamczur et al., 2015). In the present study, we observed a significant increase in the level of neuronal Glul in middle‐aged animals (Figure 8) which may point to disruption of the cross‐talk. Consequences of this alteration for synaptic plasticity are not well documented but it may be presumed that it is also a part of aging‐associated loss of the proper LTP induction.

However, it cannot be excluded that changes in hippocampal structure follow the enhanced glycolysis and increased expression of metabolic enzymes, for example, by nutritional overstimulation which leads to production of extracellular matrix and separation of hippocampal cells. It has been shown that high glucose/diabetic conditions, and hence an elevated glycolysis, may stimulate production of extracellular matrix in many tissues (peripheral nerves, heart, kidney and cancer cells (Adham et al., 2014; Ayo et al., 1991; Feng, Chen, Chiu, George, & Chakrabarti, 2008; Muona et al., 1993). It has been also shown that moderate weekly hypoglycemia prevents age‐related decline in cognitive functions (McNay, Williamson, McCrimmon, & Sherwin, 2006). Together, the above findings suggests the existence of positive feedback between glycolytic and extracellular matrix proteins expression during aging.

To conclude, one of the consequences of the observed aging‐related shift of glycolysis from the astrocyte‐associated to neuron‐based is likely to be the loss of functional LTP‐ANLS and ANLS in old mice hippocampi. In young structures, the localization of the regulatory enzyme of glycogen degradation (Pygb) and monocarboxylates transport (Mct1, Mct2) seems to corroborate the hypothesis of the classical ANLS. However, in young mice, the amount of glycolytic enzymes in hippocampal neurons is relatively high. This suggests that even there the ANLS may not be a predominant mechanism of neurons feeding. Instead, its main role may be the participation in the induction of glycogen degradation‐associated neuronal plasticity (LTP‐ANLS) and miniature excitatory postsynaptic currents, a phenomenon involved in basal synaptic transmission which deficit has been observed in hippocampi of mice strain prone to the Alzheimer disease (Fitzjohn et al., 2001).

Together, the data show that although there are evident changes in energy metabolism during aging pointing that young and old animals with energy metabolism‐related neurodegenerative disorders require different treatment, still further studies are needed to unveil precise role of these changes and to give promise of development of a new approach to treatment of brain pathologies, especially these occurring during aging.

## CONFLICT OF INTEREST STATEMENT

The authors declare no conflict of interest.

## Supporting information

Additional Supporting Information may be found online in the supporting information tab for this article.

Supporting Information

Supporting Information

Supporting Information

Supporting Information

Supporting Information

Supporting Information
