# Supplementary figures and images for: Aging‐associated changes in hippocampal glycogen metabolism in mice. Evidence for and against astrocyte‐to‐neuron lactate shuttle
Source: Glia. 2018 Mar 1;66(7):1481–95. doi: 10.1002/glia.23319 (PMC6001795; doi:10.1002/glia.23319)

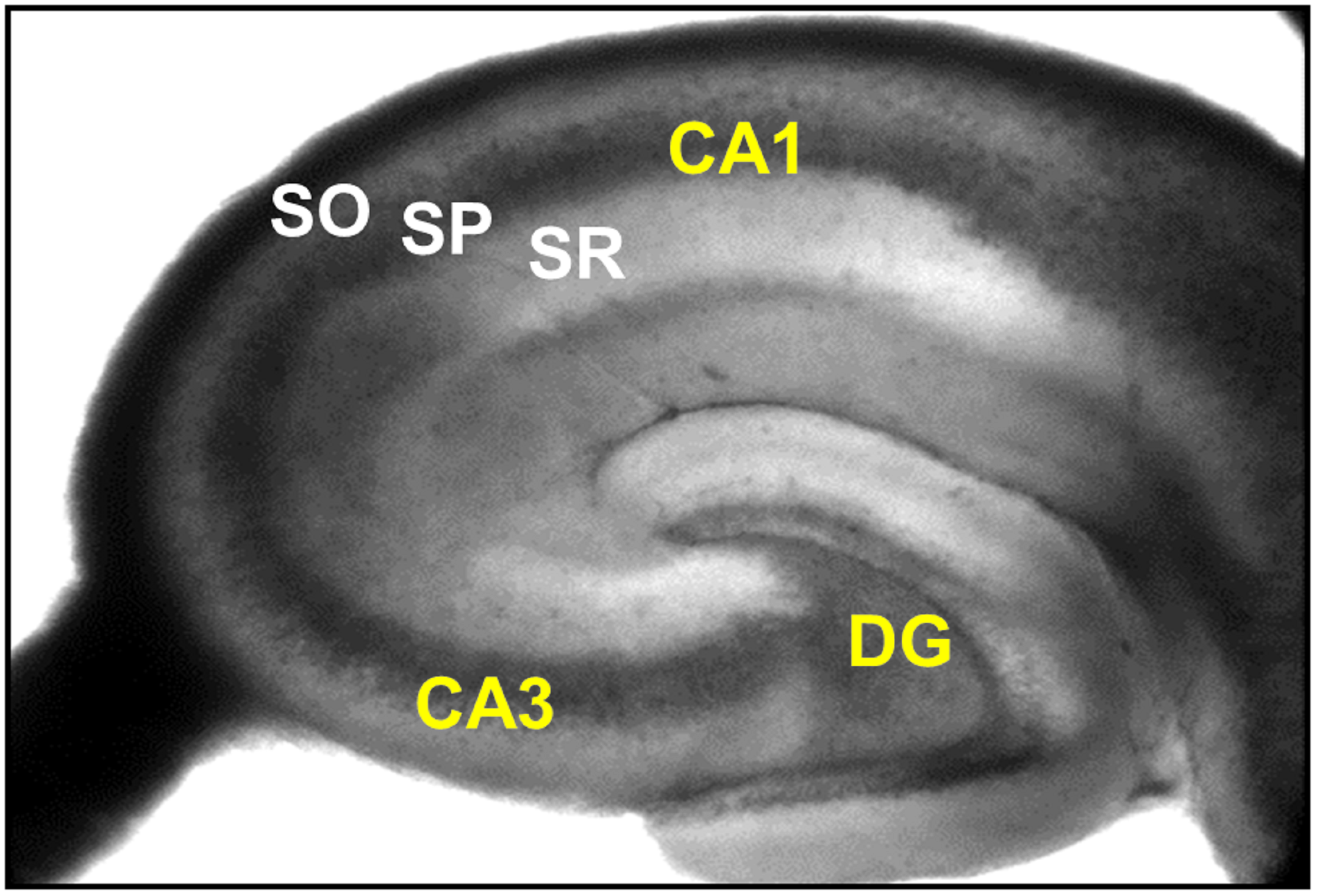

Supplement: Supplementary file 1 — Supporting Information [file GLIA-66-1481-s001.TIF]

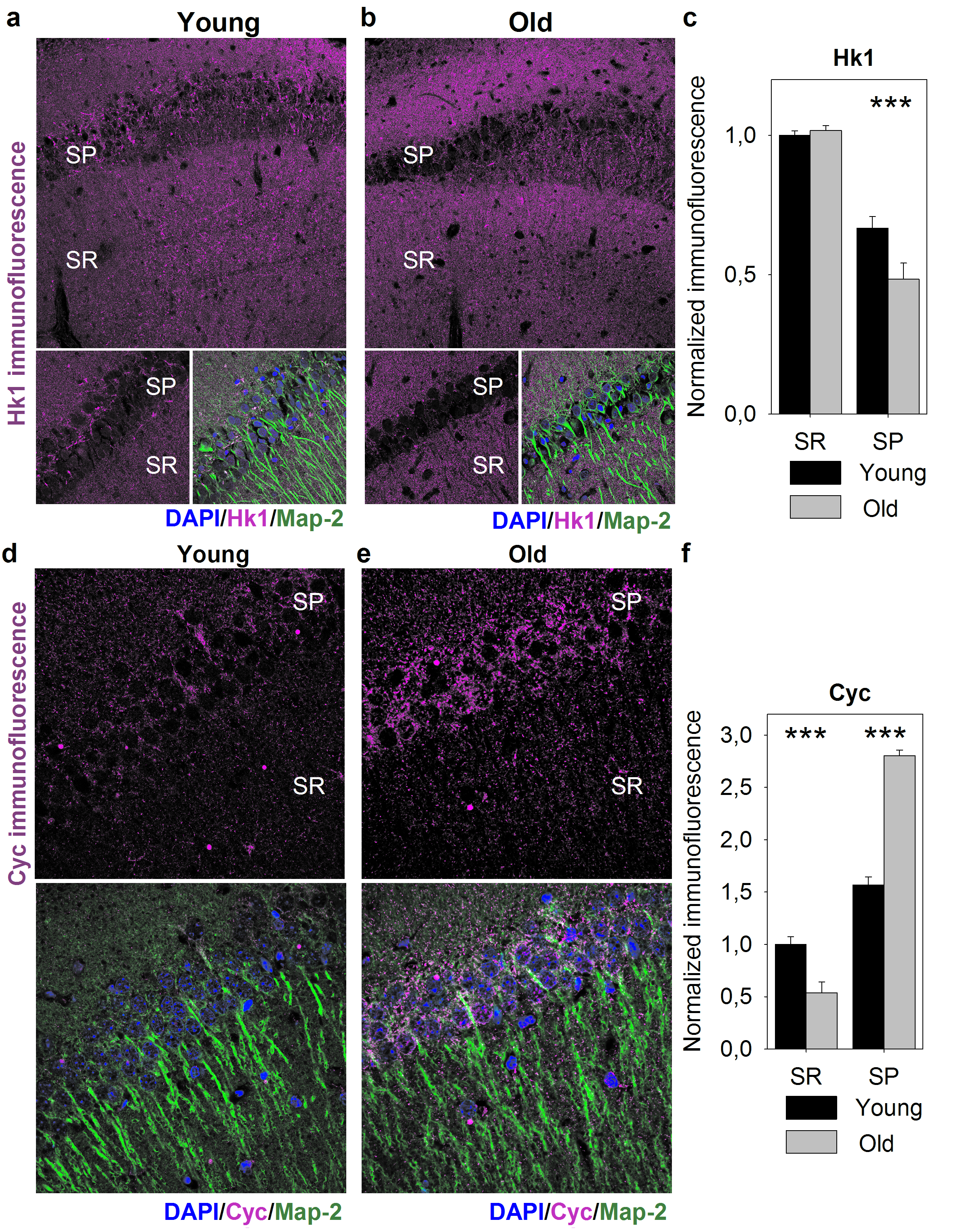

Supplement: Supplementary file 2 — Supporting Information [file GLIA-66-1481-s002.TIF]
