# Supplementary material for: Aging‐associated changes in hippocampal glycogen metabolism in mice. Evidence for and against astrocyte‐to‐neuron lactate shuttle
Source: Glia. 2018 Mar 1;66(7):1481–95. doi: 10.1002/glia.23319 (PMC6001795; doi:10.1002/glia.23319)
Supplement: Supplementary file 3 — Supporting Information [file GLIA-66-1481-s003.docx]

**Supporting Information, Table S1.** Antibodies used in the study.

| **Antigen** | **Abbreviation** | **Animal host** | **Catalog number** | **Supplier** | **Dilution** |
| --- | --- | --- | --- | --- | --- |
| **Primary antibodies to metabolic enzymes** | | | | | |
| Glycogen phosphorylase | anti-Pygb | rabbit | HPA031067 | Atlas Antibodies | 1:200 |
| Phosphoglucomutase 1 | anti-Pgm 1 | rabbit | NBP1-85982 | Novus Biologicals | 1:100 |
| Hexokinase 1 | anti-Hk1 | rabbit | ab150423 | Abcam | 1:200 |
| Phosphofructokinase platelet form | anti-Pfkp | rabbit | NBP1-19585 | Novus Biologicals | 1:100 |
| Pyruvate kinase muscle form 1/2 | anti-Pkm1/2 | rabbit | SAB4200094 | Sigma | 1:200 |
| Lactate dehydrogenase A | anti-Ldha | rabbit | NBP1-48336 | Novus Biologicals | 1:200 |
| Glutamine synthetase | anti-Glul | rabbit | G 2781 | Sigma | 1:200 |
| **Primary antibodies to metabolite transporters** | | | | | |
| **Monocarboxylate transporter 1** | anti-Mct1 (SLC16A1) | mouse | ab90582 | Abcam | 1:50 |
| **Monocarboxylate transporter 2** | anti-MCT2 (slc16A7) | rabbit | bs-3995r | STI (Bioss) | 1:50 |
| **Primary antibodies to cell markers** | | | | | |
| **Glial fibrillary acidic protein** | anti-Gfap | rabbit | G9269 | Sigma | 1:100 |
| Microtubule-associated protein 2 | anti-Map2 | mouse | M9942 | Sigma | 1:500 |
| **Secondary antibodies** | | | | | |
| anti-rabbit | AR-Alexa488 | goat | A11034 | Life Technologies | 1:2000 |
| Anti-rabbit | AR-Alexa633 | goat | A21070 | Life Technologies | 1:2000 |
| Anti-mouse | AM-FITC | goat | F8771 | Sigma | 1:500 |
| anti-mouse | AM-Alexa633 | goat | A21050 | Life Technologies | 1:2000 |
