# Supplementary material for: Aging‐associated changes in hippocampal glycogen metabolism in mice. Evidence for and against astrocyte‐to‐neuron lactate shuttle
Source: Glia. 2018 Mar 1;66(7):1481–95. doi: 10.1002/glia.23319 (PMC6001795; doi:10.1002/glia.23319)
Supplement: Supplementary file 4 — Supporting Information [file GLIA-66-1481-s004.docx]

**Supporting Information, Table S2.** TaqMan probes used for gene expression assays (Thermo Fischer Scientific).

| **protein** | ***gene*** | **TaqMan probe for gene expression assays** |
| --- | --- | --- |
| Glycogen phosphorylase | *Pygb* | Mm00464080 |
| Hexokinase 1 | *Hk1* | Mm00439344_m1 |
| Phosphofructokinase platelet form | *Pfkp* | Mm00444792_m1 |
| Pyruvate kinase | *Pkm* | Mm00834102 |
| Lactate dehydrogenase A | *Ldha* | Mm01612132-g1 |
| Lactate dehydrogenase B | *Ldhb* | Mm01267402_m1 |
| Glutamine synthetase | *Glul* | Mm00472712 |
| Glycogen synthase | *Gys1* | Mm00725701_s1 |
