# Supplementary material for: Aging‐associated changes in hippocampal glycogen metabolism in mice. Evidence for and against astrocyte‐to‐neuron lactate shuttle
Source: Glia. 2018 Mar 1;66(7):1481–95. doi: 10.1002/glia.23319 (PMC6001795; doi:10.1002/glia.23319)
Supplement: Supplementary file 5 — Supporting Information [file GLIA-66-1481-s005.docx]

**Supporting Information, Table S3.** Panel of the candidate housekeeping genes and corresponding proteins concentration detected in proteomic analysis for young and middle-aged (“Old”) animals (see Material and Methods). Most of the commonly used qRT-PCR reference genes (proteins) in our data changed during aging. We chose *Gys1* as reference for gene expression assays due to its stable protein level regardless of age (*t*-test *p* = 0.99 for young vs old animals). Asterisks indicate significant difference (* *p* < 0.05, ** *p* < 0.01, *** *p* < 0.001).

| ***Gene*** | **Protein** | **Mean Conc. [pmol/mg]**  **Young** | **Mean Conc. [pmol/mg]**  **Old** | ***t*-test result** | **direction of change** |
| --- | --- | --- | --- | --- | --- |
| *Rplp0* | 60S acidic ribosomal protein P0 | 13,80 | 10,00 | 1,66E-07 | ↓*** |
| *Rps18* | 40S ribosomal protein S18 | 18,82 | 12,63 | 6,64E-05 | ↓*** |
| *Pgk1* | Phosphoglycerate kinase 1 | 64,43 | 78,27 | 0,0014 | ↑** |
| *Gapdh* | Glyceraldehyde-3-phosphate dehydrogenase | 12,67 | 16,17 | 0,0011 | ↑** |
| *Tfrc* | Transferrin receptor protein 1 | 0,90 | 0,69 | 0,0059 | ↓* |
| *Tfrc* | Transferrin receptor protein 1 | 0,90 | 0,69 | 0,0059 | ↓* |
| *Actb* | Beta Actin | 463,00 | 428,67 | 0,0075 | ↓* |
| *Ppia* | Peptidyl-prolyl cis-trans isomerase A | 84,02 | 89,75 | 0,06 | ↑ |
| *Tbpl1* | TATA box-binding protein-like protein 1 | 0,07 | 0,11 | 0,14 | ↑ |
| *Gusb* | Beta-glucuronidase | 0,04 | 0,03 | 0,33 | ↓ |
| *B2m* | Beta-2-microglobulin | 0,04 | 0,04 | 0,87 | constant |
| *Hprt1* | Hypoxanthine-guanine phosphoribosyl transferase | 7,24 | 7,95 | 0,12 | ↑ |
| ***Gys1*** | **Glycogen synthase** | **0,40** | **0,39** | **0,99** | **constant** |
