# Supplementary material for: Aging‐associated changes in hippocampal glycogen metabolism in mice. Evidence for and against astrocyte‐to‐neuron lactate shuttle
Source: Glia. 2018 Mar 1;66(7):1481–95. doi: 10.1002/glia.23319 (PMC6001795; doi:10.1002/glia.23319)
Supplement: Supplementary file 6 — Supporting Information [file GLIA-66-1481-s006.docx]

**Supporting Information, Figure S1. Scheme of principal regions of mouse hippocampus.** View of hippocampal slice (300 µm thick) with key regions: dentate gyrus (DG), Cornus Ammonis 1 and 3 regions (CA1 & CA3). Within CA1, somata of principal hippocampal neurons (pyramidal neurons) are located primarily within striatum pyramidale (SP) and project apical dendrites to striatum radiatum (SR) while basal dendrites and axons toward stratum oriens (SO). Due to its laminar structure, CA1 region is particularly useful for localization of key neuronal structures.

**Supporting Information, Figure S2. Cellular localization of hexokinase 1 (Hk1) and cytochrome c (Cyc) in the hippocampus of young and middle-aged (”Old”) animals**. **a-b)** Exemplary confocal images of immunofluorescence distribution for Hk1 (**a, b**, magenta) within hippocampal CA1 region in young (**a**) and aged (**b**) animals acquired at low (obj. 20x, upper panel) and high magnification (obj. 60x, bottom panels). Localization of neuronal somata, dendrites and nuclei was revealed with antibodies against microtubule-associated protein 2 (Map2, green) and DAPI (blue), respectively. Abbreviations: SP- stratum pyramidale, SR-stratum radiatum. **c)** Quantification of Hk1 immunofluorescence in hippocampal slices of young (black bars) and old animals (grey bars). Note, that aging promotes reduction in Hk1 immunofluorescence exclusively in neuronal somata (SP). **d-e)** Exemplary confocal images of Cyc immunofluorescence (magenta) distribution within hippocampal CA1 region in young (**d**) and aged (**e**) animals acquired in conditions described in **a-b**.

**f)** Quantification of Cyc immunofluorescence in hippocampal slices of young (black bars) and old animals (grey bars). All immunofluorescence was normalized to values obtained within stratum radiatum of young animals. Note that in contrast to Ldha, Cyc distribution with aging tended to shit from SR to SP. Asterisks indicate a statistically significant difference (*p* < 0.001).
